# Earlier and higher dosing of alglucosidase alfa improve outcomes in patients with infantile-onset Pompe disease: Evidence from real-world experiences

**DOI:** 10.1016/j.ymgmr.2020.100591

**Published:** 2020-04-29

**Authors:** Yin-Hsiu Chien, Wen-Hui Tsai, Chaw-Liang Chang, Pao-Chin Chiu, Yen-Yin Chou, Fuu-Jen Tsai, Siew-Lee Wong, Ni-Chung Lee, Wuh-Liang Hwu

**Affiliations:** a Department of Medical Genetics, National Taiwan University Hospital, Taipei, Taiwan; b Department of Pediatrics, National Taiwan University Hospital, Taipei, Taiwan; c Department of Pediatrics, Chi Mei Medical Center, Tainan, Taiwan; d Department of Pediatrics, Cathay General Hospital, Hsinchu, Taiwan; e Department of Biological Science and Technology, National Chiao Tung University, Hsinchu, Taiwan; f Department of Pediatrics, Kaohsiung Veterans General Hospital, Kaohsiung, Taiwan; g Department of Pediatrics, National Cheng Kung University Hospital, Tainan, Taiwan; h Department of Pediatrics, China Medical University Hospital, China Medical University, Taichung, Taiwan; i Department of Medical Genetics, China Medical University Hospital, China Medical University, Taichung, Taiwan; j Department of Pediatrics, Ditmanson Medical Foundation Chia-Yi Christian Hospital, Chia-Yi, Taiwan

**Keywords:** Newborn screening, Early treatment, Enzyme replacement therapy, Pompe disease, Dosage, CK, creatine kinase, CRIM, cross-reactive immunological material, ERT, enzyme replacement therapy, GAA, acid alpha-glucosidase, GMFM, Gross Motor Function Measure, IOPD, infantile-onset PD, ITI, immune tolerance induction, NBS, newborn screening, PD, Pompe disease, PDMS-2, Peabody Developmental Motor Scale, Second Edition, uGlc4, urine glucose tetrasaccharide

## Abstract

**Objective:**

Enzyme replacement therapy (ERT), the only approved therapy for infantile-onset Pompe disease (IOPD), had heterogeneous clinical effects due to factors such as severity, age at first treatment, dosage, and dosing regimens. We report the clinical and biochemical outcomes of a cohort of IOPD patients identified through newborn screening, and evaluating the dosage effect.

**Study design:**

A retrospective observational study was designed to describe the long-term clinical and biochemical outcomes of a uniform cohort of IOPD patients who have been treated with high-dosage of ERT.

**Results:**

Twenty-eight patients received alglucosidase alpha at either the labeled dosage followed by a high dosage (*n* = 23) or a high dosage exclusively (*n* = 5). At a median age of 8.3 years (0.8–17.3), 15 patients were walkers, 8 were weak walkers, and 5 were nonwalkers. The three groups exhibited a significant difference in the age of gross motor decline (*p* < .001). In patients with classical IOPD diagnosed through newborn screening, those late in ERT initiation (*p* = .006) or late in high-dosage ERT initiation (*p* = .044) had a higher risk of motor decline. At the latest assessment, both serum creatine kinase (CK) and urinary glucose tetrasaccharide (uGlc4) levels were lowest in the walkers. During follow up, the biomarker levels, once rose, never returned to normal.

**Conclusion:**

Low CK and uGlc4 levels were correlated with favorable response to ERT in IOPD patients, although CK may be more fluctuated than uGlc4. High-dose ERT instituted immediately at newborn screening seems to give the best outcome, and a dosage increase is necessary upon – or, even better, before – a rise in biomarker levels.

## Highlights

• CK and uGlc4 levels were correlated with favorable response to ERT in IOPD patients.
• Once these biomarkers rose, they never returned to normal.
• High-dose ERT instituted immediately at newborn screening seems to give the best outcome.

## 1 Background

Pompe disease (PD), a rare autosomal recessive condition also known as glycogen storage disease type II, is caused by a deficiency of the lysosomal enzyme acid alpha-glucosidase (GAA). The most severe form, classic infantile-onset PD (IOPD), typically features dramatic hypertrophic cardiomyopathy at birth, while atypical (non-classical) IOPD can present cardiomyopathy several months later. Enzyme replacement therapy (ERT) with recombinant human GAA (Myozyme®, alglucosidase alpha) is the only treatment currently available. ERT effectively reverses cardiomyopathy, improves motor development, and improves overall survival [[1], [2], [3]]. Both the introduction of ERT at a young age [4] and a low titer of anti-rhGAA antibodies [5] are critical to achieve a good treatment outcome. We previously demonstrated that patients diagnosed through newborn screening were treated very early and presented more favorable outcomes than patients who were diagnosed by clinical symptoms [6].

Unfortunately, all long-term survivors of IOPD, including those in our newborn screening cohort, showed residual myopathy [7] which led to failure to achieve motor milestones [8,9]. Several studies suggested that patients may benefit from an ERT dosage higher than the labeled doses [[10], [11], [12], [13], [14]], but the dosage, frequency, timing, and indication for using a higher dosage were variable in these studies. Given this variability combined with the heterogeneity in the severity of IOPD cases and the ages of patients at ERT initiation, it is difficult to draw a conclusion about the use of high-dose ERT.

Taiwan has had a newborn screening program for Pompe disease since 2005, and we followed all patients detected by prospective monitoring. All patients identified in the current study were positive for cross-reactive immunological material (CRIM), and most of them had a low titer of anti-rhGAA antibodies. This homogeneous cohort of IOPD patients is the best context in which to evaluate the efficacy of different ERT regimens. Here, we present our experience with 28 IOPD patients receiving different ERT dosages.

## 2 Materials and methods

**Fig. 1:**
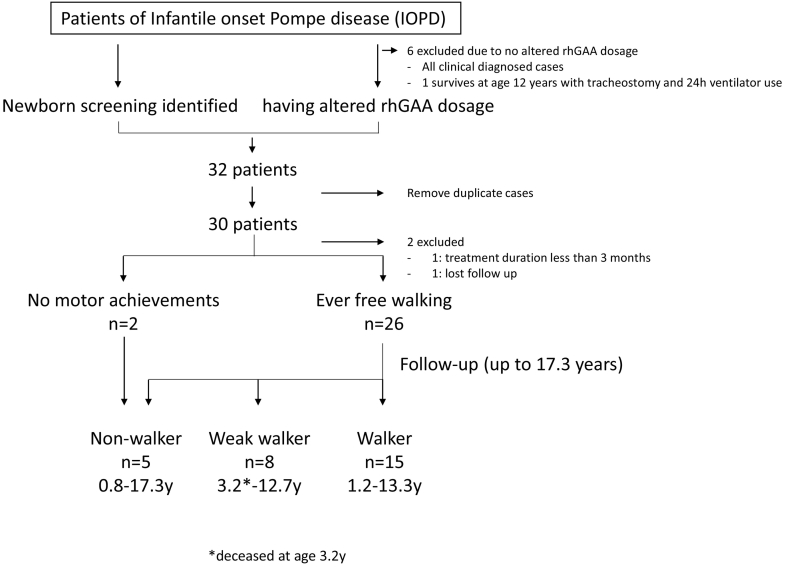
Patient flow and outcome of IOPD patients concerning current motor function.

A retrospective multicenter observational study was designed to describe the long-term clinical and biochemical outcomes of a cohort of IOPD patients, involving seven centers and enrolled patients born in the period from Jan 2002 to Dec 2019. The inclusion criteria for the patients were as follows: a) identification by the Newborn Screening (NBS) programs, confirmed diagnosis of IOPD; b) confirmed diagnosis of IOPD after symptom onset, followed at some point in time by the use of high-dosage ERT. After duplicate cases were removed, 30 patients (17 females, 13 males) were enrolled for further analysis (Fig. 1). Patients who confirmed the diagnosis of IOPD after symptom onset without altering the ERT dosage were excluded from the study. All patients were presenting Pompe disease relating symptoms/biochemical abnormalities in infancy. Patients presenting with significant cardiomegaly at birth were defined as classical IOPD. Patients presenting cardiomegaly at a later age [15] or no cardiomegaly were defined as atypical or non-classical IOPD.

For patients who have been treated in our hospital, a prospective follow-up protocol has been in place since 2008 [6]; therefore a longitudinal data of biochemical and motor function outcomes were available for this study. For all patients, data concerning age at diagnosis, age at ERT initiation, signs and symptoms at disease onset and during the follow-up visits, achieved motor milestones, motor function assessments (Gross Motor Quotient (GMQ) of the Peabody Developmental Motor Scale, Second Edition (PDMS-2), Gross Motor Function Measure (GMFM)-88 E domain), and respiratory function (need for ventilator support) were collected. PDMS-2 provides data relevant to the norm, for example, GMQ being expressed as percentile of norm. GMFM-88, on the other hand, provides information regarding to the child's own development. Parameters concerning the treatment included dosage/frequency of ERT, age at the time of any dosage/frequency changes, adjuvant medicines such as albuterol [16], immune tolerance induction (ITI) therapy, and the use of other formulations of recombinant human GAA. Laboratory data, including *GAA* mutation, serum creatine kinase (CK), urine glucose tetrasaccharide (uGlc4), and IgG antibodies against rhGAA (performed by Genzyme Clinical Specialty Laboratory), was collected. Urinary glucose tetrasaccharide (uGlc4) concentrations were determined using the ultra-high-performance liquid chromatography (UHPLC) tandem mass spectrometry (MS/MS) method [16,17], and the age-appropriate normal range was established for comparison. The age of gross motor decline was defined as the age of GMQ equal to or lower than the 5th percentile in patients from birth to 5 years of age, or the age of 6-min walk test *Z*-score ≤ −2 in patients age 5 years or older [18]. Data from patients using a different formulation of ERT were excluded from this study. Informed consent was obtained from all parents or their legal representatives on behalf of patients.

### 2.1 Statistics

We collected data from patients for more than 17 years, and the dosages varied among patients. Therefore, we also calculated the lifelong (average) dosage for each patient. Both 40 mg/kg every other week (eow) and 20 mg/kg weekly (qw) were considered a double dose and assigned a multiplier of 2, although they may not be equal on pharmacokinetics or immunogenicity. For patients taking the labeled dosage, their lifelong average dosage was 1. If patients spent 1/7 of the time taking 40 mg/kg eow and took the labeled dosage the rest of the time, their lifelong dosage would be 1.14 (6/7 + 1/7*2 = 1.14). The results are presented as medians with ranges. Correlations between two variables were calculated using Pearson's correlation coefficient. Group differences were analyzed using the Mann-Whitney *U* test or the Kruskal-Wallis test with post hoc analysis. A general linear model with post hoc LSD test was used for mutivariate analysis. *Kaplan-Meier* curve and Cox regression analysis were used to evaluate predictors of age of gross motor decline, including sex, and the variables of treatments. Statistical analyses were performed with SPSS 17.0 (SPSS, Armonk, NY). The significance level was set at 0.05 for all tests.

## 3 Results

### 3.1 Patient characteristics

Table 1 shows the demographics, genetics, and clinical data before ERT initiation, as well as methods of therapeutic management (ERT dosage and ITI) of the 30 patients. Six IOPD patients diagnosed after symptoms onset from our hospital were excluded due to no dosage change, and only one of them survived with invasive ventilation now at age 12 years. A few patients (Nos. 1–4, 6–17) have been mentioned partially in previous clinical outcome publications [3,6,7,15,[19], [20], [21]]. The most common *GAA* mutation, c.[1935C > A;1726G > A] (p.[D645E; G576S]), was noted in 35 (58%) of the total 60 alleles. Three (10%) patients did not have this common mutation, but they were also CRIM positive. The median baseline serum CK level was 759 IU/L (94–5767), and the baseline uGlc4 was 26.17 mmol/mol Cre (13.61–62.24; *N* < 12.9). All but one patient received alglucosidase alpha starting before they reached 6 months of age [22 (73%) before 1 month of age). The median age of ERT initiation was 0.5 months for the newborn-screened cohort (*n* = 24) and 4.5 months for the clinically identified cohort (*n* = 6). In the first 24 patients (80%), the initial ERT dosage was 20 mg/kg eow, while the following 6 patients received 40 mg/kg eow starting from the beginning of ERT due to the treatment policy changed. Two patients were excluded from the outcome analysis because one refused to be followed, and the other had an ERT duration of less than 3 months (Fig. 1).

Among the 28 patients included in the data analysis, 23 patients had initially received the labeled dosage. Twenty-two (96%) of those 23 patients had their ERT dosages increased at a median age of 35 months (6–114) due to clinical deterioration or biochemical data change. The dosages were either increased to 30 mg/kg eow and then 40 mg/kg eow, or directly to 40 mg/kg eow or 20 mg/kg qw. Although 20 mg/kg qw was preferred dosage because several patients reported deterioration during the biweekly ERT, parents could choose 40 mg/kg eow for their convenience. For patients who experienced no benefit from a higher dosage, the dosage could be decreased to the labeled dose or lower. Five patients started ERT at a dosage of 40 mg/kg eow, and among them, 2 (40%) had a further increase in dosage (at 8 months in one case and 18 months in the other). The median lifetime ERT dosage in all patients was 1.58 (1.00–2.27) times the labeled dosage. Twenty-seven of the 28 patients were alive at the end of data collection, with a median age of 8.3 years (0.8–17.3). The median most recent CK level of the survivors was 1092 IU/L (83–1832), and their uGlc4 level was 27.44 mmol/mol Cre (4.41–91.11; age-appropriate normal <7).

**Supplemental Fig. 1:**
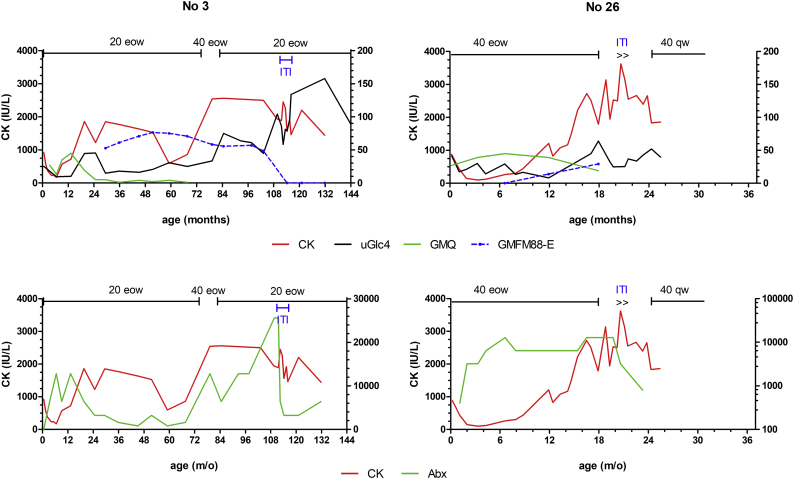
Time courses of (upper panel) serum CK, uGlc4, Gross Motor Quotient (GMQ) of PDMS-2, GMFM-88 E domain scores, and management (ERT, immune induction therapy (ITI)); (lower panel) serum CK and anti-rhGAA antibodies (Abx) for 2 IOPD patients with intermediate antidrug antibodies. CK is plotted on the left Y-axis, while the other 4 values are plotted on the right Y-axis. 20 eow: 20 mg/kg every other week; 40 eow: 40 mg/kg eow; 40 qw: 40 mg/kg every week.

Patient No. 26 experienced severe reactions including massive urticaria during ERT, which was managed by premedications (corticosteroids and antihistamine) and a slow and very diluted infusion for 12 h. She and patient No. 3, both identified through newborn screening, had intermediate antibody titers (1:12800 to 1:25600), such that ITI was applied using a protocol provided by Dr. Priya Kishnani. After one course of ITI, the antibody titers decreased from 1:25600 to 1:3200 in No. 3 and from 1:12800 to 1:800 in No. 26. Unfortunately, both patients demonstrated elevation of biomarker levels, and No. 3 gradually lost her motor function (Supplemental Fig. 1).

None of the patients required oxygen supplementation or ventilator support at ERT initiation (baseline). At the latest assessment, one patient (No. 1) had a tracheostomy and was ventilator dependent. There were 5 patients who needed to use noninvasive ventilators at night due to hypoventilation. The baseline motor function assessments in the newborn screening group revealed normal results. During the follow-up period, all but two patients (No. 1 and No. 5) learned to walk. One patient (No. 13) died suddenly at the age of 3.3 years; at that time, she could walk with assistance. Over time, some patients lost motor milestones due to progressive muscle weakness; these patients were classified based on the current status as nonwalkers (Nos. 1–5, *n* = 5) or weak walkers (Nos. 6–13, *n* = 8). The others (Nos. 14–28, *n* = 15) were classified as walkers.

### 3.2 Variables determining responses to treatment

Overall, older patients tend to have a delay in the time of first high-dosage ERT (correlation coefficient (C.C.) 0.775, *p* < .001) and higher latest uGlc4 levels (C.C. 0.565, *p* = .002). The most recent CK level was positively correlated with the latest uGlc4 level (C.C. 0.475, *p* = .012).

**Fig. 2:**
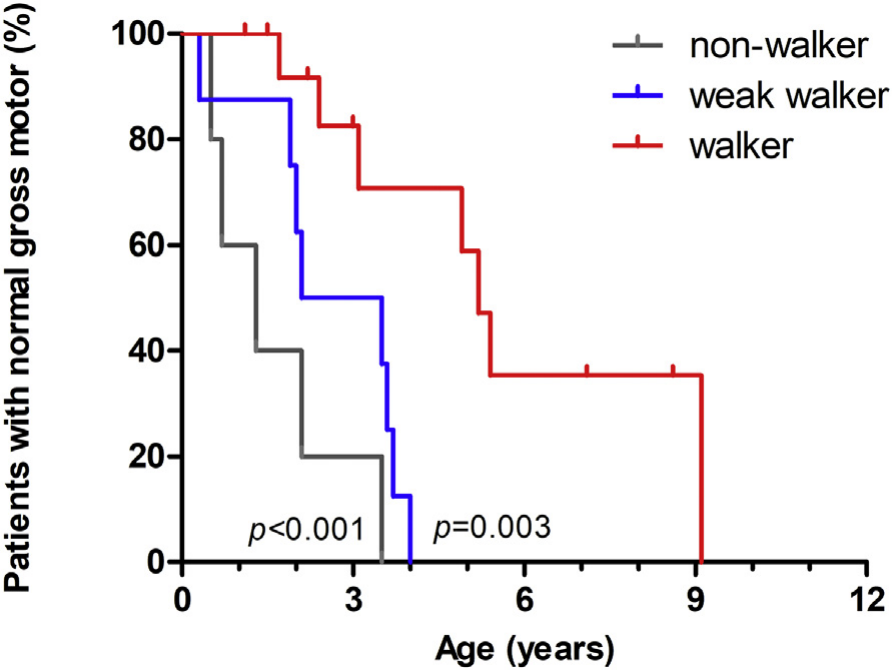
Kaplan-Meier analysis of the age of gross motor function decline in patients. The event was defined as Gross motor quotient equal to or lower than the 5th percentile in patients from birth to 5 years of age, or the age of 6-min walk test *Z*-score ≤ −2 in patients age 5 years or older. Patients were grouped into nonwalkers, weak walkers, and walkers according to the current status.

**Fig. 3:**
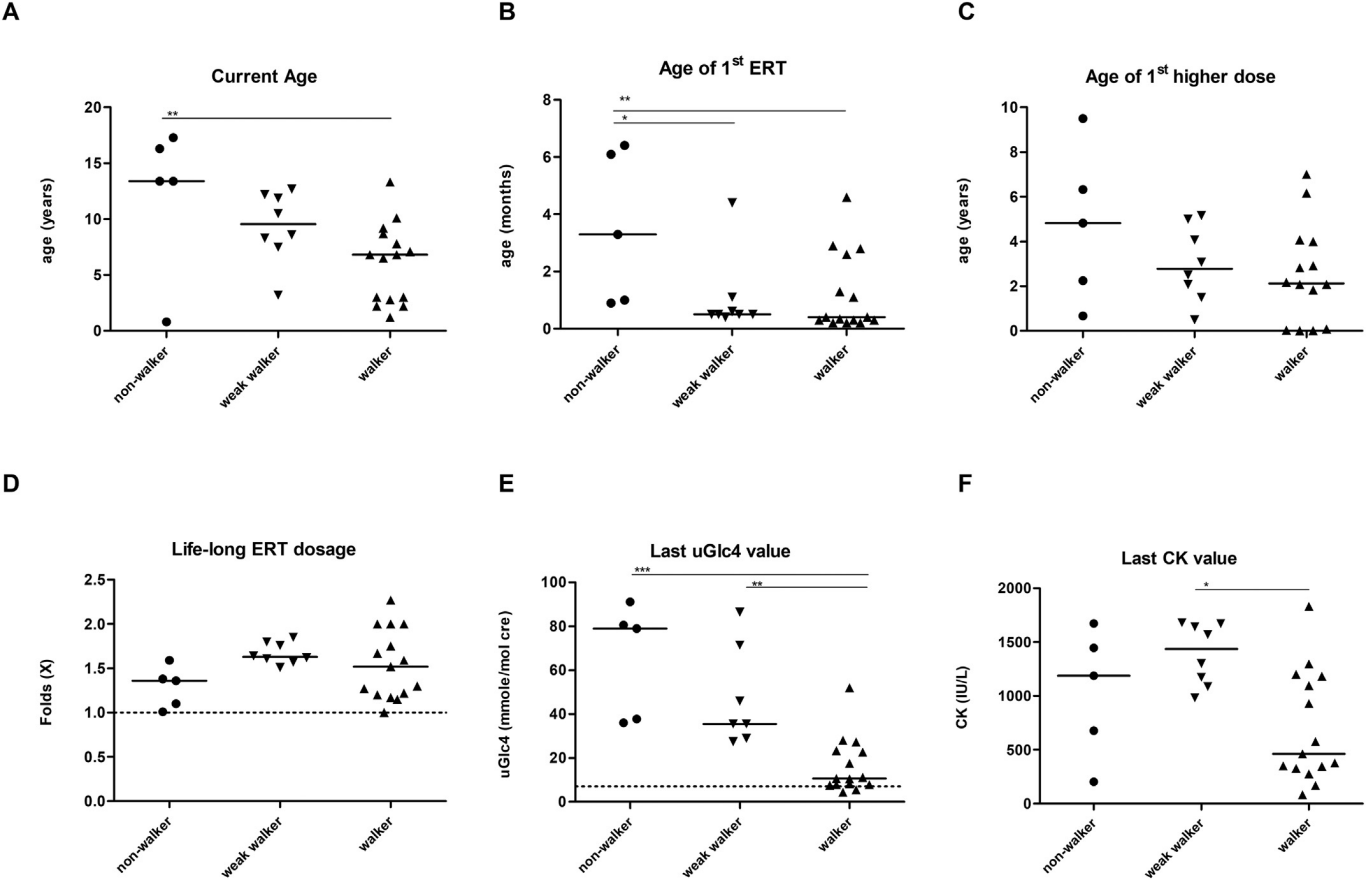
Current age (A), age of ERT initiation (B), age of 1st high dose (C), lifetime ERT dosage (D), and biomarkers urine Glc4 (E) and serum CK (F) at last assessment in all patients. Patients were grouped into nonwalkers, weak walkers, and walkers. * indicates *p* < .05; ** *p* < .01; *** *p* < .001.

We compared multiple variables for the nonwalker, weak walker, and walker groups. The three groups showed a significant difference in the time of gross motor function decline (*p* < .001) (Fig. 2). The walker group could be separated from the nonwalker (*p* < .001) and the weak walker (*p* = .003), approximately starting from the age of 3 years. The median age of high-dosage ERT initiation in the nonwalker, weak walker, and walker group was 4.8, 2.8, and 2.1 years, respectively. Multivariate analysis of variance (MANOVA) revealed that the nonwalker group was the oldest (*p* = .017) and with late ERT initiation (*p* = .024), but the age at introduction of high-dosage ERT (*p* = .332) and the average lifelong ERT dosage (*p* = .124) were not significantly different among the three groups (Fig. 3). None of these factors affect the risk of gross motor function decline in Cox regression model.

**Fig. 4:**
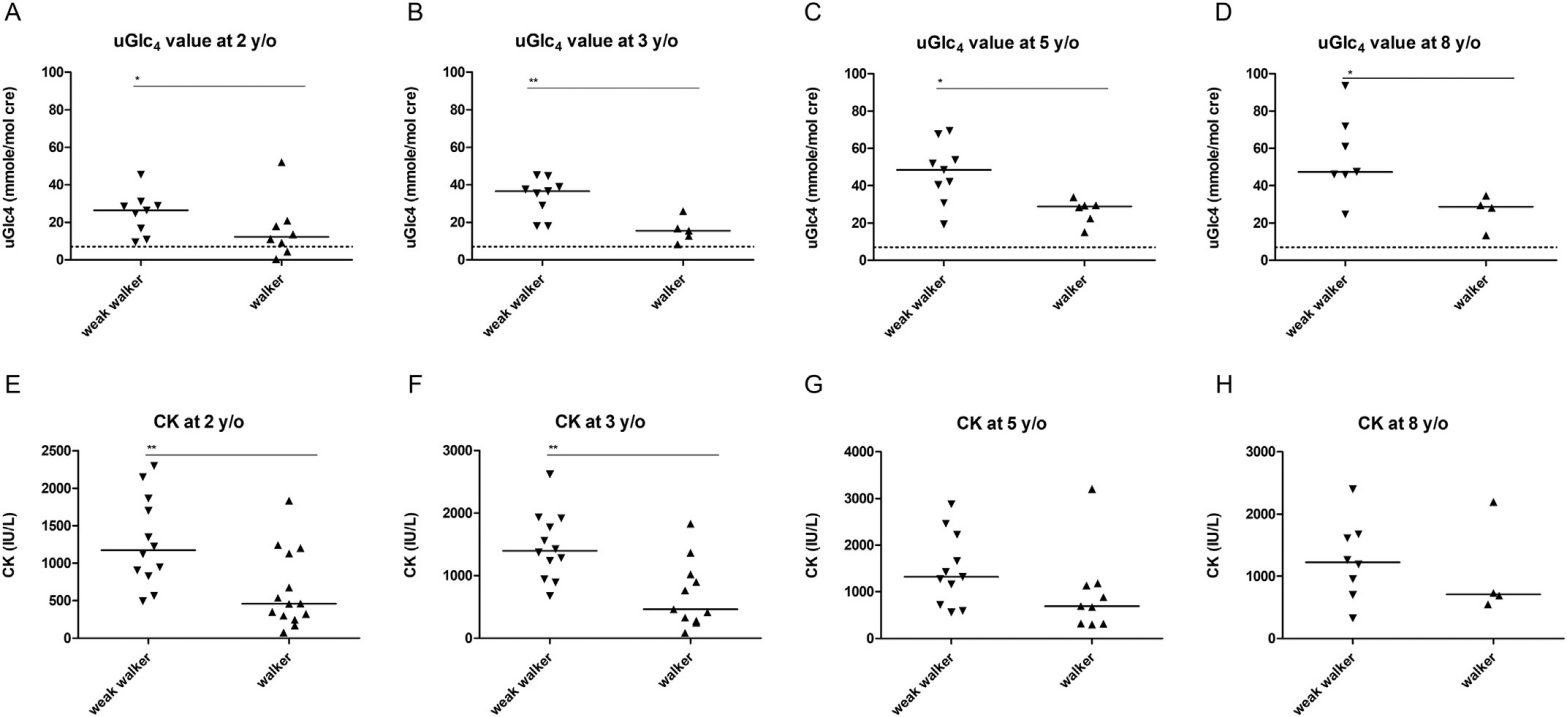
Urine Glc4 (A-D) and serum CK (*E*-H) by year in all patients. Patients were grouped into weak walkers (including nonwalkers and weak walkers) and walkers. Compared to the weak walker group, walkers have reduced uGlc4 levels at ages 2 through 8 years and reduced CK levels at ages 2 and 3 years. * indicates *p* < .05; ** *p* < .01; *** *p* < .001.

Both biomarkers, CK and uGlc4, were compared through the follow-up period. At baseline, CK and uGlc4 were similar among groups. At the most recent assessment, the walkers had lower uGlc4 levels (*p* < .001, Fig. 3E) and lower CK levels (*p* = .027, Fig. 3F) among all patients. Since the walker group was younger than the weak walker group, we further compared the levels at specific ages. The walkers had lower uGlc4 levels at 2 years (*p* = .023), 3 years (*p* = .004), 5 years (*p* = .012), and 8 years of age (*p* = .042; Fig. 4A-D) than the weak walkers (including nonwalkers). The walkers also had lower CK levels than the weak walkers at 2 years (*p* = .005) and 3 years of age (*p* = .004).

We further extracted newborn-screening-identified patients with cardiomegaly at birth, the classic IOPD (11 walkers and 9 weak walkers (including 2 nonwalkers)), for further comparison as two groups. The median age of high-dosage ERT initiation in the weak walkers (and nonwalkers), and walker group was 4.0, and 2.0 years, respectively. Multivariate analysis revealed that the weak walker group was older than the walker group (*p* < .001). Overall, late in ERT initiation (Hazard Ratio (HR) 267; 95% CI: 4.876–14,710; *p* = .006) and late in high-dosage ERT initiation (HR 3.057; 95%CI: 1.031–9.064; *p* = .044) were risks for gross motor function decline in this classical IOPD newborn screening group.

**Fig. 5:**
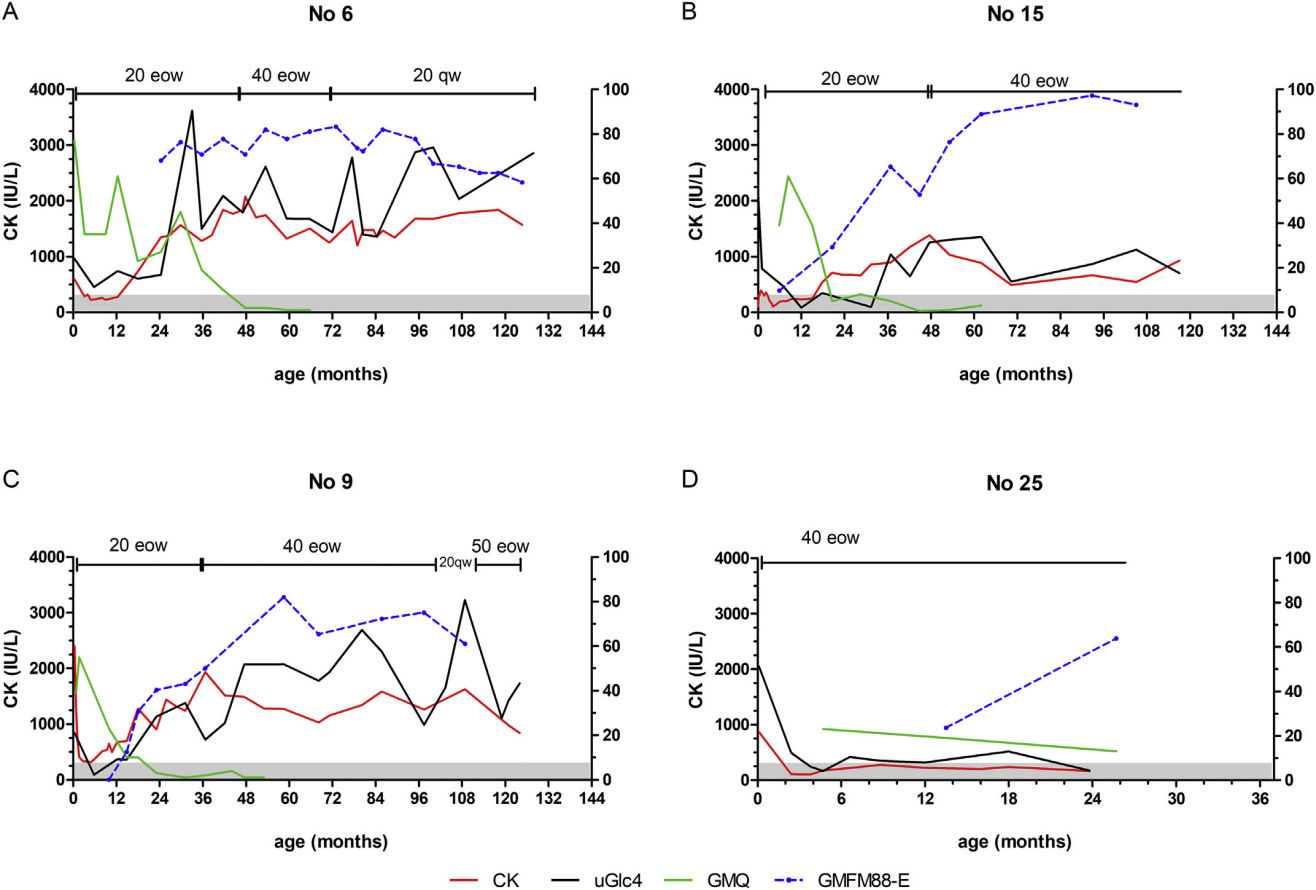
Characteristic time courses of serum CK, uGlc4, Gross Motor Quotient (GMQ) of PDMS-2, scores of GMFM-88 E domain, and management (ERT dosage and frequencies) for 4 IOPD patients. Patients starting with 20 mg/kg eow who showed initial improvement in CK and uGlc4 (A-C). These biomarkers rebounded after 6 months of age, and the ERT dosage was increased. The biomarkers became stationary (A) or even decreased (B) with higher ERT dosage, or benefited minimally from the current higher dose (C). In contrast, patient identified through newborn screening and started on 40 mg/kg eow showed completely normalized CK and uGlc4 (D) till age 2 years. CK was plotted on the left Y-axis, while the other 3 values are plotted against the right Y-axis. 20 eow: 20 mg/kg every other week; 40 eow: 40 mg/kg eow; 20 qw: 20 mg/kg every week. The scale of the X-axis was adjusted for the relatively short duration of their follow-up (D).

**Supplemental Fig. 2:**
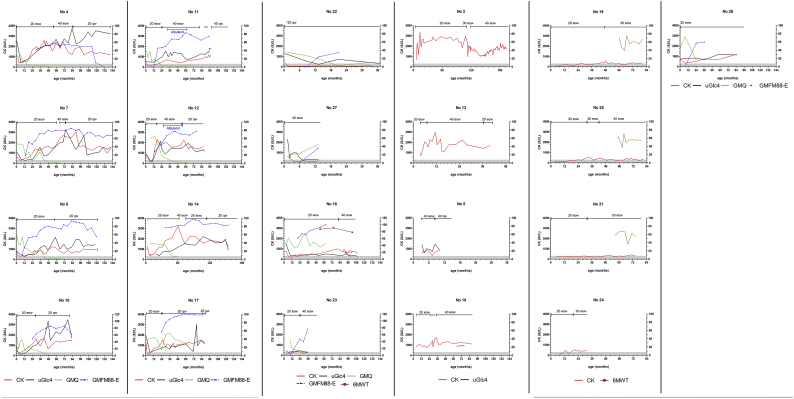
Time courses of serum CK, uGlc4, Gross Motor Quotient (GMQ) of PDMS-2, scores of GMFM-88 E domain, % of prediction of 6-min walk test, and management (ERT dosage and frequencies) for 21 IOPD patients. Patient No. 1 was not spotted for no complete detail of serum CK during these years. CK was plotted on the left Y-axis, while the other 4 values are plotted against the right Y-axis. 20 eow: 20 mg/kg every other week; 40 eow: 40 mg/kg eow; 20 qw: 20 mg/kg every week.

We observed uGlc4 levels changed in different ways in response to ERT dosage (Fig. 5, Supplement Fig. 2). In the patients who started with the labeled dosage (Fig. 5 A-C), uGlc4 and CK levels decreased in the first 6–12 months of ERT and then rose in all cases. The dosage of ERT changed only after the decline of motor function (poor performance by GMQ) (Fig. 5 A-C). However, the decline in motor function comparing to the norm, as shown by low percentile of GMQ, was not apparently on the GMFM-88 E domain, as the patients still have motor gain for another 3–5 years (Fig. 5 B-C). Increasing the ERT dosage may stabilize (Fig. 5 A), decrease but not to normal uGlc4 (Fig. 5B), or do not affect uGlc4 (Fig. 5C). uGlc4 act similar to the serum CK levels, but serum CK reacted faster than uGlc4 at the beginning years. In contrast, patients who received 40 mg/kg eow beginning from ERT initiation (Fig. 5 D) retained normal uGlc4 and CK levels, and their motor function was also normal at their latest follow-up.

## 4 Discussion

Although ERT above the labeled dose has been suggested to improve the outcome of IOPD patients, few studies can prove it because of the high heterogeneity of the patients and treatment conditions. In the current study, we analyzed the effect of high-dose ERT using a cohort of prospectively followed IOPD patients who were homogeneous in genotype, CRIM status, and time of ERT initiation because they were detected by newborn screening. We employed parameters including motor scales and the biomarkers CK and uGlc4. In our analysis, older patients tend to have higher uGlc4, especially in the weak walker (including nonwalker) patients. The individual time course showed the motor response, serum CK and uGlc4, while patients in good motor function were tending to remain low serum CK and uGlc4. We demonstrated a benefit to patients who received high-dose ERT either presymptomatically or before any clinical or biochemical signs of deterioration.

The two keystone clinical trials [2, 3] provide useful reference for the benefit of higher ERT dosages. Van den Hout et al. first demonstrated that muscle GAA activity returned to normal 12 weeks and glycogen concentration was partially normal 84 weeks after 40 mg/kg qw of rhGAA rabbit milk origin [2]. Another rhGAA, Myozyme®, tested in both 20 mg/kg eow and 40 mg/kg eow [3], showing a higher increase in skeletal muscle GAA activity (median 137 nmol/h/g tissue compared to 70.1 nmol/h/g tissue) and more significant reductions of glycogen in skeletal muscles (median decrease 75.0% compared to 45.2%) [3] in the 40 mg/kg group. In addition, the duration for which uGlc4 remained low was longer in the 40 mg/kg group than that in the 20 mg/kg group in the good response group (Group A) [22,23]. The fact that uGlc4 but not CK remained low in the good response group [22,23] emphasizes the value of uGlc4 in the prediction of treatment response. The new developments for Pompe disease ERT, such as avalglucosidase alpha [24] and ATB200/AT2221 [25], also target a higher dosage than the current labeled dose of alglucosidase alpha.

The beneficial effect of a dosage increase was suggested in a number of reports, but the effects remained inconclusive. For patients experiencing motor decline, reports showed that an increased dose was beneficial in some patients [8,26], but other reports found otherwise [9]. A high starting dosage of 40 mg/kg qw was shown to completely correct muscle damage in a small trial with 5 patients [13,27]. In the current study, we demonstrated that biomarker changes occurred earlier than clinical deterioration, and the response to high-dose ERT varied, but in all patients, high-dose ERT needs to be instituted before the occurrence of biochemical changes. The effect of high-dose ERT in our study, only stabilize/limit the progression of disease but not normalize the biochemical changes, in patients who already exhibit clinical deterioration, probably due to not optimize the doses in our previous practice. Only with high-dose ERT (at least double of the labeled dose) starting immediately after newborn screening, as shown in the current study and a single case report by Spada et al. [27], can CK and uGlc4 levels stay normal. The uptake of recombinant GAA depends on the mannose-6-phosphate (M6P) receptor, and the expression of the receptor changes with age [28]. Lysosomal glycogen storage can result in dysfunction of autophagosomes, which will diminish the recycling of the M6P receptor and consequently, the uptake of rhGAA [29]. Once this vicious cycle starts, even high-dose ERT will no longer be effective.

We observed no apparent differences in safety parameters among different ERT dosages. Certainly, therapeutic proteins can induce an immune response neutralizing the effect of ERT. Two patients, one receiving the labeled dose and the other receiving 40 mg/kg eow, developed persistent intermediate-high antibody titers. All patients, regardless of CRIM status or dosage, should have regular antibody monitoring.

There were several limitations in this observation. First, the learning process during these years introduced several different ERT dosages and frequencies, the changes being based on the experience but not a prospective study. However, the younger patients, starting ERT earlier and on a higher dose earlier, showed a delay in the motor decline ages. Second, the follow-up protocol, although a prospective in nature, may not have CK, uGlc4 and motor evaluation at the same time. In addition, the motor evaluations, GMFM88, PDMS-2, and 6MWT, may not be correlated perfectly to the clinical outcomes for the wide-range of ages covered in this study. Nevertheless, we were the first to the best of our knowledge to contribute to the largest systemic real-world data in IOPD. Third, we only included patients clinically identified with the experience of higher ERT dosage. Other clinically identified patients, under the labeled dosage, were poor responders and were excluded.

In conclusion, low serum CK and uGlc4 levels correlated with the clinical response of IOPD patients to ERT, although CK may be more fluctuated than uGlc4. A dosage increase is suggested immediately or even before biomarker levels rise, but the maximal dosage is currently unclear. We suggest that a high dosage of ERT such as 40 mg/kg eow as the starting dosage, may be more effective than the labeled dose in stabilizing and improving the clinical prognosis of patients with IOPD even if they were identified through newborn screening. Close monitoring of biomarkers and antibody titers as well as planning for an individualized dosage may further improve clinical outcomes.

The following are the supplementary data related to this article.

## Funding

Sanofi/Genzyme partially sponsored the study of the long-term follow-up (GZ-2014-11166) and immune tolerance induction (GZ-2015-11336). The study sponsor had no involvement in 1) study design; 2) the collection, analysis, and interpretation of data; 3) the writing of the report; and 4) the decision to submit the manuscript for publication. YHC wrote the first draft of the manuscript. None of the authors received whether an honorarium, grant, or other form of payment to produce the manuscript.

## Declaration of Competing Interest

**Yin-Hsiu Chien** has served on advisory boards for Amicus Therapeutics and Sanofi Genzyme, undertaken contracted research for Sanofi Genzyme, received honoraria, consulting fees, and travel expenses from Sanofi Genzyme.

**Wuh-Liang Hwu** has served on advisory boards for Audentes and Sanofi Genzyme, undertaken contracted research for Sanofi Genzyme, received honoraria, consulting fees, and travel expenses from Sanofi Genzyme.

**Ni-Chung Lee** has served on Registry advisory boards for Sanofi Genzyme, received honoraria and travel expenses from Sanofi Genzyme.

**Wen-Hui Tsai** has served as a site principal investigator for the Rare Disease Registry Program and received honoraria and travel expenses from Sanofi Genzyme.

**Chaw-Liang Chang, Pao-Chin Chiu, Fuu-Jen Tsai, Yen-Yin Chou**, and **Siew-Lee Wong** declare no conflict of interest.
